# Integrative Transcriptome Analysis Across Follicles Highlights Key Regulatory Pathways in Low and High-Egg-Laying Hens

**DOI:** 10.3390/ani15223300

**Published:** 2025-11-15

**Authors:** Armughan Ahmed Wadood, Farhad Bordbar, Xiquan Zhang

**Affiliations:** 1 State Key Laboratory of Swine and Poultry Breeding Industry, Guangzhou 510640, China; armughanwadood@gmail.com (A.A.W.); Farhadnevergiveup@yahoo.com (F.B.); 2 Guangdong Provincial Key Lab of Agro-Animal Genomics and Molecular Breeding, Key Lab of Chicken Genetics, Breeding and Reproduction, Ministry of Agriculture and Rural Affair, South China Agricultural University, Guangzhou 510642, China

**Keywords:** chicken, folliculogenesis, transcriptomics, steroidogenesis, granulosa cells, egg-laying

## Abstract

Egg-laying performance in hens is regulated by complex molecular mechanisms within the hypothalamic–pituitary–gonadal (HPG) axis and ovarian follicles. This study employed integrative transcriptome profiling of primordial (PR), primary (PM), small white (SW), and small yellow (SY) follicles in hens with low and high egg-laying capacities to explain regulatory pathways influencing reproductive outcomes. Specific gene expression patterns were observed that correlated with follicular growth, steroidogenesis, and granulosa cell proliferation. Heatmap clustering and principal component analysis revealed transcriptional divergence between low- and high-laying hens, suggesting that coordinated changes in signaling pathways influence egg-laying performance. High-laying hens intricated an upregulation of the PI3K-AKT-FOXO3, TGF-β, and Wnt/β-catenin pathways, which facilitate early follicular development, granulosa cell proliferation, and folliculogenesis. Higher phosphorylation of AKT and reduced nuclear FOXO3 activity were associated with enhanced primordial follicle growth. Increased TGF-β signaling, as demonstrated by higher levels of SMAD2/3/4 and cell cycle regulators, promoted granulosa cell proliferation in primary follicles (PMF). In SWF, higher levels of β-catenin and its downstream genes, such as c-Myc and cyclin D1, promoted follicle development. High-laying hens revealed increased expression of FSHR, CYP19A1, 17β-HSD, CYP1A1, and CYP1B1 in SYF, signifying enhanced FSH level and steroidogenesis. Similarly, low-laying hens exhibited downregulation of key genes, suggesting reduced follicular development and hormone signaling. These findings identify key regulatory networks and molecular markers associated with reproductive performance, providing targets for genetic selection and interventions to enhance egg production while reducing the risk of hormonal overstimulation.

## Simple Summary

This research investigates the molecular mechanisms that influence egg-laying performance in hens by analyzing transcriptome profiles of follicles at various developmental stages in hens with differing egg-laying capacities. The study analyzes primordial (PR), primary (PM), small white (SW), and small yellow (SY) follicles to identify essential gene expression patterns associated with follicular growth, steroidogenesis, and granulosa cell proliferation. The study identifies notable transcriptional variations between low- and high-laying hens. High-laying hens show upregulation of critical signaling pathways, including PI3K-AKT-FOXO3, TGF-β, and Wnt/β-catenin, which facilitate early follicular development and granulosa cell proliferation. Furthermore, high-laying hens exhibited improved steroidogenesis and folliculogenesis, as shown by the elevated expression of genes associated with follicle-stimulating hormone (FSH) signaling. Conversely, low-laying hens exhibited downregulation of these pathways, correlating with diminished follicular development and hormone signaling. The findings enhance comprehension of the genetic and molecular foundations of egg-laying performance and identify potential targets for interventions in poultry breeding programs.

## 1. Introduction

Egg production in chickens is a highly regulated biological process, with ovarian folliculogenesis playing a key role in influencing egg-laying efficiency [1]. Follicles advance through multiple developmental stages, from PR to SYF, each defined by unique morphological and molecular characteristics [2,3]. Understanding the molecular pathways that regulate follicular development and their impact on egg-laying capacity remains a considerable challenge in avian research [4,5,6]. This study examines transcriptomic differences between ovarian follicles in chickens with high and low egg-laying capacity to identify markers of reproductive performance.

The ovarian follicles in chickens undergo a precisely regulated process of development and maturation, culminating in ovulation [7]. Complex interactions among hormones, growth factors, and underlying genetic instructions regulate this process [8]. Follicular development in hens is categorized into several stages, ranging from PRF to PMF, small SWF, SYF, and large yellow follicles, each defined by unique gene expression patterns [9,10]. During follicular development, coordinated interactions among molecular signaling pathways facilitate the growth and survival of the dominant follicle, while the others undergo atresia [11,12]. The regulatory mechanisms governing which follicles progress to ovulation and which regress are not fully defined [13]. Identifying these pathways could provide key insights into the genetic regulation of egg-laying efficiency [14,15].

Recent advances in RNA sequencing (RNA-Seq) technology enable the investigation of gene expression across tissues and developmental stages [16,17]. At different stages of ovarian follicular development, a transcriptomic study reveals gene expression variability [18]. Several studies have identified genes and mechanisms involved in follicular growth and maturation; however, they have primarily focused on specific follicles or phases [19,20,21]. Integrative investigations that incorporate follicular development and compare chickens with different reproductive outcomes are necessary to understand the molecular pathways of egg-laying [22,23]. We employed an integrated transcriptome analysis of ovarian follicles from hens with low and high egg-laying rates to elucidate mechanisms regulating egg production.

This study examines gene expression profiles to identify molecular signatures that distinguish high-laying chickens from those with low reproductive efficiency. We hypothesized that high-laying chickens exhibit distinct gene regulatory networks governing reproduction. We propose that high-laying chickens exhibit distinct gene regulatory networks and signaling pathways to optimize follicular development, ovulation, and egg production. High- and low-laying birds must be molecularly distinguished to improve poultry breeding. Biomarkers associated with high egg-laying efficiency may enable the development of selective breeding and therapeutic approaches to enhance hen egg production. The transcriptional landscape of follicular development will reveal the molecular mechanisms of oocyte quality, atresia, and hormone regulation. Transcriptomic research can uncover egg-laying pathways; however, few studies have investigated the complex gene networks and regulatory processes that distinguish high- and low-laying hens. This discovery may explain egg-laying efficiency, fowl reproductive regulation, and industrial egg production at the molecular level.

## 2. Materials and Methods

### 2.1. Animals and Sample Collection

This study included healthy adult laying hens of the Chinese Xinghua chicken (*Gallus gallus domesticus*) with low and high egg-laying performance, aged 60 to 65 weeks, and maintained under typical poultry house settings. Birds were euthanized via cervical dislocation, in compliance with institutional animal care and ethical standards. All experimental procedures involving female chickens in this article were approved by the Institutional Animal Care and Use Committee, South China Agricultural University, with the approval ID SCAU#2025f235. All experiments were conducted in accordance with the established regulations and guidelines. After euthanasia, the ovaries were removed, immersed in ice-cold phosphate-buffered saline (PBS), and conveyed to the laboratory for further processing.

### 2.2. Follicles Classification and Isolation

**Figure 1:**
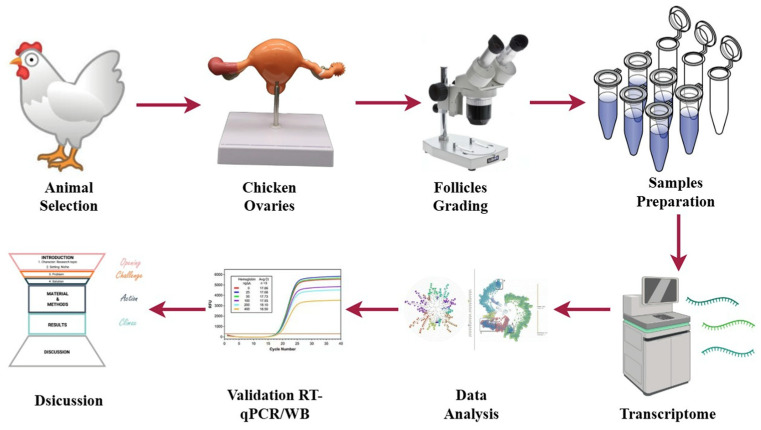
Graphical representation of the experimental workflow for transcriptomic analysis in low- and high-egg-laying hens.

Follicles were carefully dissected with a stereomicroscope and categorized into four developmental stages based on size, pigmentation, and morphology: PRF (PF, <80 µm, distinguished by a single layer of flattened granulosa cells), PRF (PR, 80–1000 µm, exhibiting cuboidal granulosa cells), SWF (SWF, 2–4 mm, translucent), and SYF (SYF, 4–6 mm, yellow-pigmented). Follicles from three different animals were combined for each category to create biological triplicates (Figure 1). A total of twenty-four (*n* = 24) hens were used in this study, comprising twelve biological replicates per group (High-laying, *n* = 12; Low-laying, *n* = 12). For each biological replicate, RNA was extracted from the follicles of a single, individual hen. Therefore, each data point represents an independent biological sample.

### 2.3. RNA Extraction and Quality Assessment

Using the TRIzol reagent (Invitrogen, Carlsbad, CA, USA) and following established methods, total RNA was isolated from each stage of the follicle. Follicle tissues were homogenised in TRIzol, and RNA was extracted using a phase separation method with chloroform, followed by isopropanol precipitation and ethanol washing. A solution of RNase-free water was used to resuspend the RNA pellets. A NanoDrop spectrophotometer (Wilmington, DE, USA) was used to assess RNA purity and concentration, and an Agilent 2100 Bioanalyzer (Santa Clara, CA, USA) was used to validate RNA integrity. The selected samples for sequencing had RIN values greater than 7.0.

### 2.4. Transcriptome Library Preparation and RNA Sequencing

RNA samples from each follicular group, with three replicates each, were used to construct sequencing libraries using the NEBNext Ultra RNA Library Prep Kit from New England Biolabs (Ipswich, MA, USA). Messenger RNA was isolated using oligo(dT) magnetic beads, then fragmented, yielding the first and second complementary DNA (cDNA) strands. Subsequent steps included end repair, adaptor ligation, and PCR amplification. The library’s quality and quantity were evaluated using Qubit fluorometry and Bioanalyzer analysis. Sequencing was performed on the Illumina NovaSeq 6000 platform, producing 150 bp paired-end reads and an average of 40 million clean reads per sample.

### 2.5. Transcriptome Data Processing and DEG Analysis

Raw sequencing data were initially assessed using FastQC v0.12.1 and then processed with Trimmomatic to remove low-quality reads and adaptors. Clean reads were aligned to the Gallus gallus reference genome (GRCg6a) using HISAT2. Transcript assembly and expression quantification were performed using StringTie v2.2.2, with gene expression assessed in FPKM units. Differentially expressed genes (DEGs) were identified through DESeq2, applying a threshold of |log2 fold change| ≥ 1 and an adjusted *p*-value < 0.05. Gene expression patterns were examined using Venn diagrams, hierarchical clustering heatmaps, volcano plots, and principal component analysis (PCA) in R.

### 2.6. Gene Ontology and KEGG Pathway Enrichment

The ClusterProfiler package in R (R 4.5.2) was used to perform functional enrichment analysis of differentially expressed genes (DEGs). Gene Ontology (GO) terms are classified into three primary categories: biological processes (BP), molecular functions (MF), and cellular components (CC). The Kyoto Encyclopedia of Genes and Genomes (KEGG) analysis indicated enriched pathways during follicular transitions. Significance was evaluated using a Benjamini–Hochberg-adjusted *p*-value threshold of <0.05. The enrichment results were illustrated using bar plots, distinguishing between upregulated (red) and downregulated (blue) gene categories across developmental stages.

### 2.7. Protein–Protein Interaction (PPI) and Hub Gene Identification

Regulatory genes were identified by inputting differentially expressed genes (DEGs) into the STRING database to construct protein–protein interaction (PPI) networks. Network files were visualised and analysed in Cytoscape (v3.9). Hub genes were identified using the cytoHubba plugin, which employed degree centrality as the criterion. Three transition-specific networks were established for the transitions from PF to PRF, PMF to SWF, and SWF to SYF. Nodes with the highest connectivity were designated as hub genes, indicating their critical functions in follicular regulation (as of 30 August 2025).

### 2.8. Signaling Pathway Modeling

Three primary signalling pathways were reconstructed based on transcriptomic and enrichment analyses: the COL6A1-PTEN-PIP3-AKT pathway, active during the transition from PRF to PMF; the Wnt/β-catenin-TCF/LEF pathway, active during the PRF-to-SWF transition; and PLB1-mediated glycerophospholipid metabolism, which regulates the shift from SWF to SYF. The signalling pathways were illustrated using BioRender and diagrams.net, utilising KEGG data and relevant literature to represent the dynamic regulation of folliculogenesis (as of 29 August 2025).

### 2.9. Descriptive Statistics

Descriptive statistics were computed to summarize the central tendency and variability of egg-laying data for both low and high egg-laying groups. The mean and standard deviation (SD) were calculated for each group to estimate central tendency and data dispersion, respectively. The standard error of the mean (SEM) was calculated to quantify the precision of the mean values. The S.E.M. was employed to evaluate the reliability of the observed mean differences among the groups. Statistical calculations were conducted using standard software (e.g., Excel 2024, R, or SPSS V.30), with results presented as mean ± S.E.M. (assessed on 30 August 2025).

### 2.10. qRT-PCR Validation of DEGs

Quantitative real-time PCR (qRT-PCR) was performed on selected differentially expressed genes (DEGs) to validate the transcriptomic data, with an emphasis on key signaling and metabolic pathways. Total RNA from each group was reverse transcribed using the PrimeScript RT reagent kit (Takara, Japan) [24]. Quantitative reverse transcription polymerase chain reaction (qRT-PCR) was conducted using SYBR Green PCR Master Mix (Applied Biosystems) on a StepOnePlus Real-Time PCR system. Primer sequences were designed using Primer-BLAST v.4.1.0 and evaluated for amplification efficiency. Expression data were normalized to β-actin and analyzed using the 2^−ΔΔCt^ method [25]. All reactions were performed in triplicate, and statistical analysis was conducted using ANOVA followed by Tukey’s post hoc test, with *p* < 0.05 considered significant.

## 3. Results

### 3.1. Transcriptome Data Analysis

**Figure 2:**
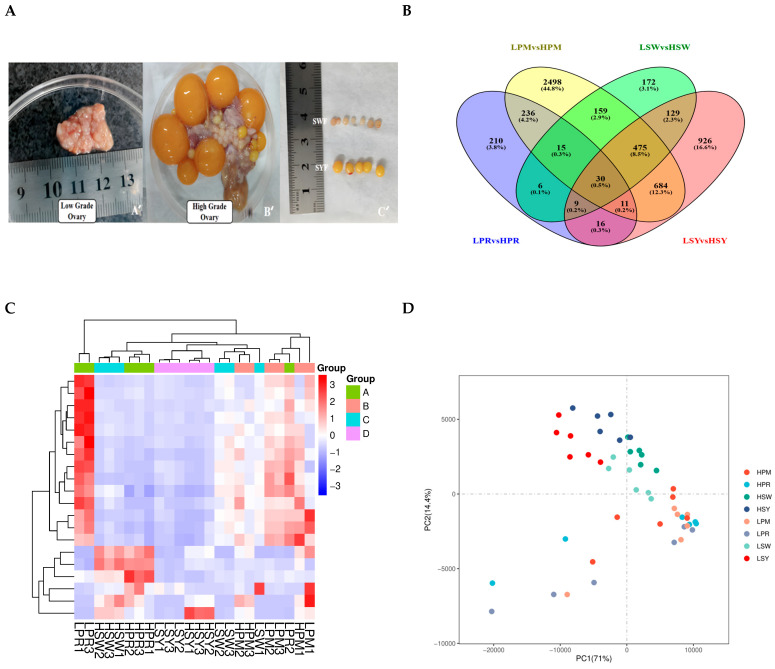
(**A**–**D**) Phenotypic and transcriptomic analysis of ovarian follicles in low- and high-laying hens. Representative images of low-grade and high-grade laying hen ovarian tissue illustrating follicle growth morphology: (**A**) High-grade ovaries exhibit a clearly defined yolky follicle structure (**B’**), whereas low-grade ovaries (**A’**) display less distinct structures. Dissected follicles were classified from SW to SY for transcriptome analysis (**C’**). (**B**) A Venn diagram illustrates the distribution and overlaps of differentially expressed genes (DEGs) in the PRF, PMF, SYF, and SYF follicle phases of low- and high-egg-laying hens. All follicular stages exhibited 30 differentially expressed genes (DEGs). (**C**) The heatmap illustrates the differentially expressed genes (DEGs) across all samples, revealing distinct expression patterns associated with follicle types and laying performance groups. Colour intensity represents Z-score-normalised expression levels. (**D**) The PCA map indicates that samples are differentiated based on follicle stage and egg-laying performance. The initial two principal components account for most of the variation, suggesting biological differences particular to the groups.

This study conducted a thorough investigation to clarify molecular differences among follicle groups in hens with varying egg-laying capacities. Figure 2A illustrates the classification and grading of chicken follicles, delineating clear distinctions between low- and high-grade follicles, with graphic representations of PRF, PMF, and SYF, each classed according to morphology and dimensions. Figure 2B illustrates a Venn diagram that emphasizes the convergence of transcriptomic profiles among four distinct follicle categories—low to high PR, PM, SW, and SYF—showcasing considerable overlap and unique gene expression within these groups, which may suggest regulatory mechanisms influencing variations in egg-laying (Table 1).

**Figure 3:**
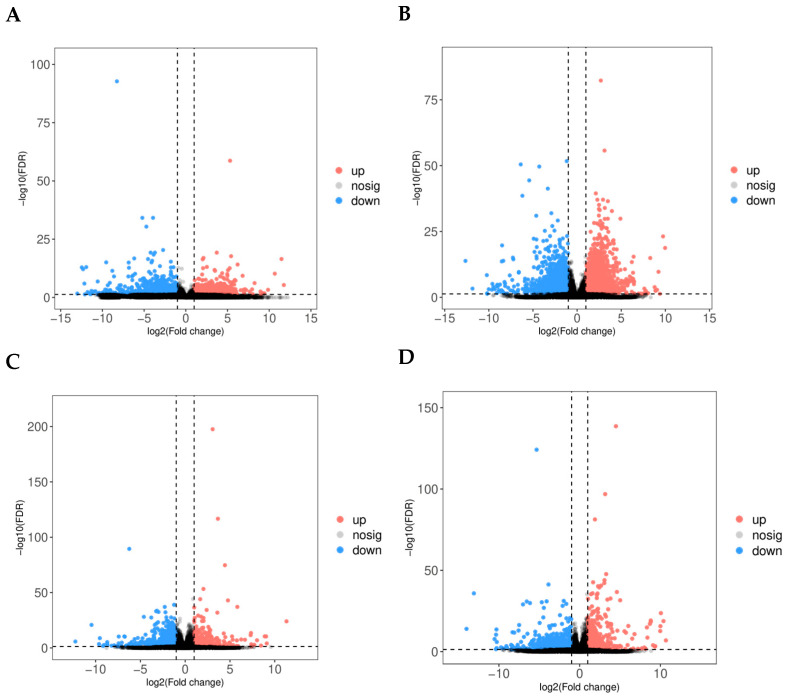
(**A**–**D**) Volcano plots display transcriptomic changes in (**A**) LPR vs. HPR, (**B**) LPM vs. HPM, (**C**) LSW vs. HSW, and (**D**) LSY vs. HSY follicles. The *x*-axis represents the log2 fold change (high vs. low), and the *y*-axis represents the −log10 of the false discovery rate (FDR). Significantly upregulated genes (FDR < 0.05, log2FC > 0) are shown in red, downregulated genes (FDR < 0.05, log2FC < 0) are shown in blue, and non-significant genes are shown in grey.

The heatmap (Figure 2C) corroborates these findings, showing varying gene expression patterns among the follicle groups, and hierarchical clustering reveals discrete groupings of follicles based on their transcriptomic profiles. The PCA analysis (Figure 2D) emphasises heterogeneity in gene expression along the principal components (PC1 and PC2), indicating intrinsic differences in follicular transcriptomes related to egg-laying potential. Principal component analysis revealed a clear transcriptomic separation between high- and low-laying groups. Notably, we observed greater heterogeneity within the high-laying group. These figures collectively highlight the significance of follicle-specific gene regulation in egg-laying chickens, offering critical insights into the regulatory pathways that may influence egg production efficiency. Additional data are presented in Supplementary Tables S1–S19, which include genes and corresponding protein accession numbers (DEGs) associated with follicular development. Transcriptome analysis identified a significant number of genes that are differentially expressed in high versus low egg-laying chickens. The volcano plot (Figure 3A–D) demonstrates a distinct separation, with many transcripts exhibiting significant differential expressions at a threshold of |log2(Fold Change)| > 1 and a *p*-value < 0.05. The analysis of individual comparisons uncovered unique groups of differentially expressed genes (DEGs): 532 for LPR vs. HPR, 4107 for LPM vs. HPM, 994 for LSW vs. HSW, and 2279 for LSY vs. HSY. A comprehensive analysis of these DEGs resulted in a unique collection of 3666 genes. In this combined dataset, 1437 genes exhibited significant upregulation, while 2229 genes showed downregulation in the high egg-laying group relative to the low egg-laying group. This unique transcriptional profile indicates significant underlying differences in biological processes associated with egg production.

### 3.2. Gene Ontology Enrichment Analysis

The GO enrichment analysis of differentially expressed genes (DEGs) across the four follicular stages, PRF, PMF, SWF, and SYF, identified distinct and stage-specific biological functions related to follicular development in high and low egg-laying hens. During the initial phases (PRF and PMF), the upregulated genes were primarily enriched in Gene Ontology (GO) terms associated with cellular processes, metabolic processes, and biological regulation within the biological process (BP) category. Additionally, these genes showed significant catalytic activity and binding in the molecular function (MF) category, as well as in cell and organelle components in the cellular component (CC) category.

**Figure 4:**
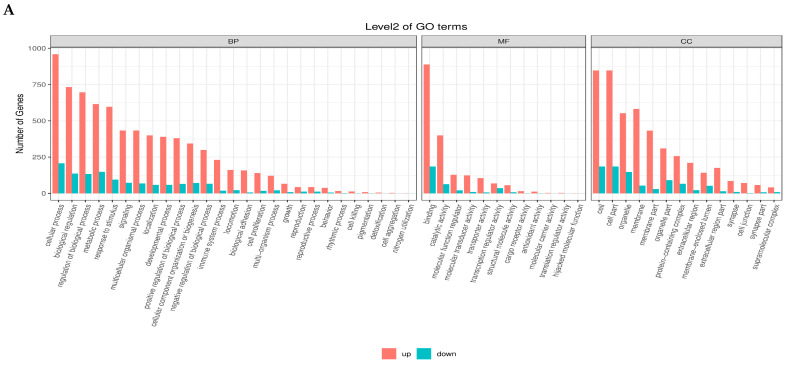

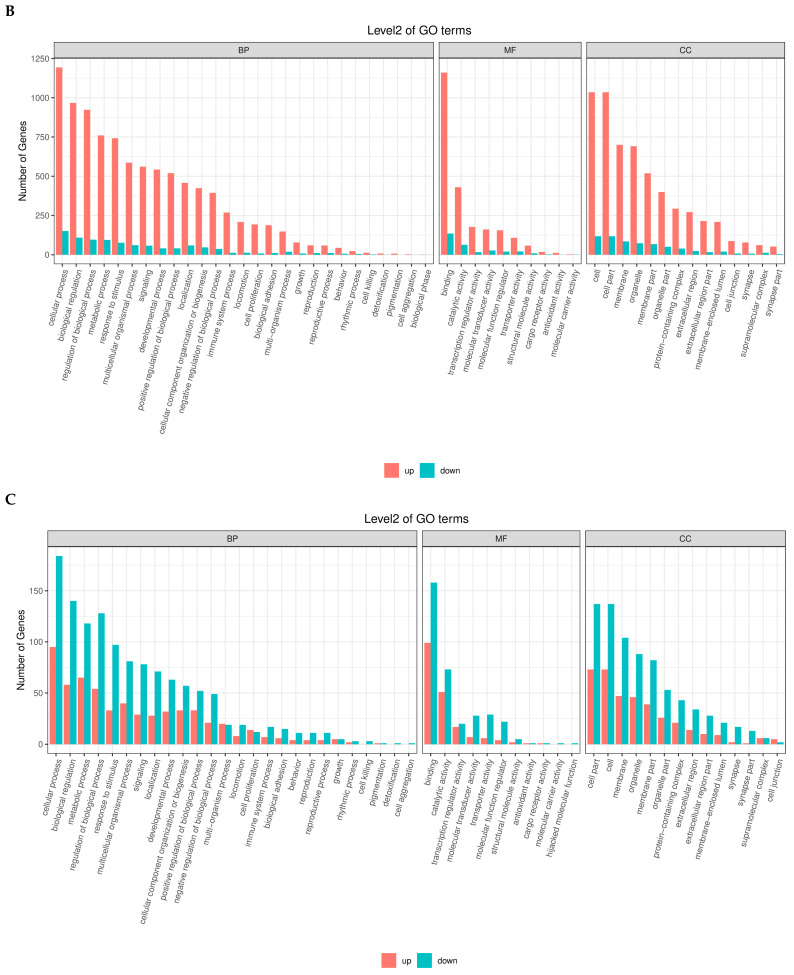

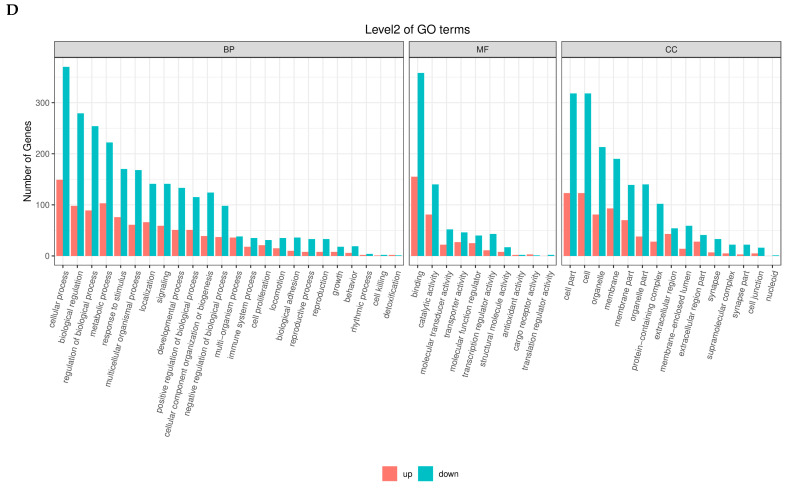
(**A**–**D**) Gene Ontology (GO) enrichment analysis of differentially expressed genes (DEGs) across four follicular stages, PRF, PMF, SWF, and SYF in chicken ovaries, contrasting low- and high-egg-laying hens. The bar plots illustrate the distribution of upregulated (red) and downregulated (blue) differentially expressed genes (DEGs) across the three primary Gene Ontology (GO) domains: biological process (BP), molecular function (MF), and cellular component (CC). Each subplot depicts the principal GO terms enriched within the appropriate domain, with counts reflecting the number of DEGs linked to each phrase. These comparison profiles show significant changes in gene regulatory activity, implying essential biological processes and pathways associated with follicular growth and their possible influence on egg-laying ability.

During the progression from SW to SYF, a significant shift occurred, characterised by the prominence of downregulated genes, particularly in pathways related to structural molecules, signal transduction, and cellular communication. High-egg-laying hens exhibited an increased number of upregulated genes linked to reproductive and metabolic processes, suggesting improved follicular activity and regulatory mechanisms. In contrast, hens with low egg production showed greater downregulation in essential functional categories, suggesting diminished transcriptional activity during folliculogenesis. The results show dynamic gene regulation throughout the follicle development stages and suggest that variations in gene expression patterns between high- and low-egg-laying hens may contribute to differences in reproductive performance (Figure 4A–D).

### 3.3. KEGG Enrichment Analysis

**Figure 5:**
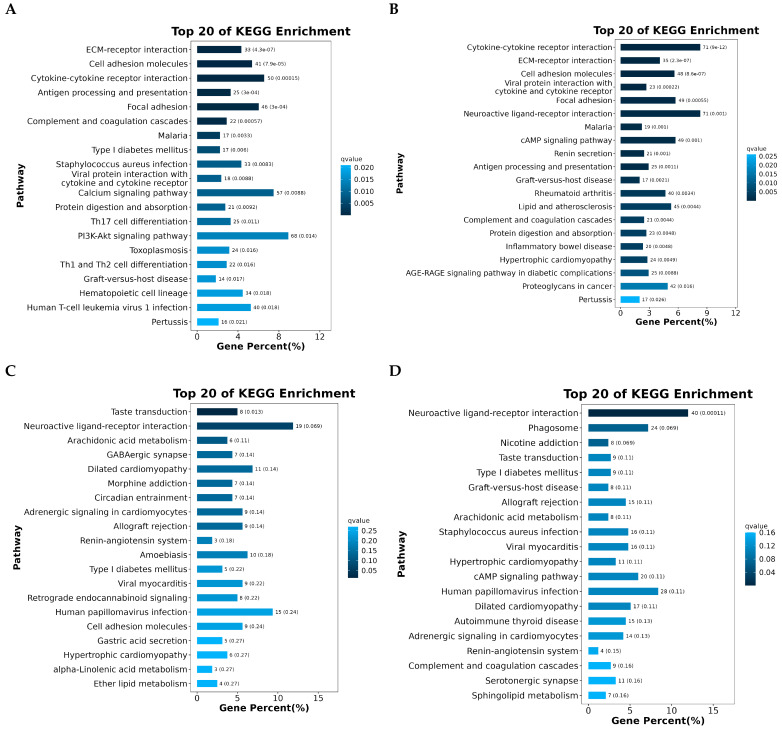
(**A**–**D**) KEGG pathway enrichment analysis was conducted on differentially expressed genes (DEGs) across four stages of follicular development—PR, PM, SW, and SYF—in chicken ovaries, comparing high- and low-egg-laying hens. The bar charts depict the top 20 significantly enriched pathways, ordered by gene percentage and differentiated by adjusted q-value colour coding. Enriched pathways across stages encompass immune response mechanisms, such as cytokine–cytokine receptor interactions and antigen processing and presentation; cell communication processes, including ECM–receptor interactions and focal adhesion; and metabolic pathways, notably arachidonic acid and sphingolipid metabolism. The differences in pathway enrichment across follicular stages demonstrate stage-specific transcriptional dynamics, highlighting the importance of immune signaling, metabolic regulation, and intercellular signaling pathways in follicle development and egg production potential in hens.

The KEGG enrichment analysis of differentially expressed genes across the four follicular stages revealed dynamic, stage-specific activation of essential biological pathways involved in follicle development and reproductive performance in chickens. In the initial phases, pathways highly enriched in PRF and PMF included immune-related interactions, such as cytokine–cytokine receptor interactions, ECM–receptor interactions, and antigen processing and presentation, indicating a significant role of immune signaling in early follicular activation. During development from SW to SYF, a substantial enrichment of metabolic and signaling pathways was observed (Figure 5A–D).

This included pathways related to neuroactive ligand–receptor interactions; arachidonic acid metabolism; and hormone-related signalling, such as the cAMP and renin–angiotensin pathways. Pathways related to disease and immune regulation, such as graft-versus-host disease and viral infections, were consistently observed across stages, highlighting the interplay between immune readiness and follicular maturation. The changes in enriched pathways are a shift from immune-modulated early development to metabolically and hormonally driven later stages. This highlights the molecular differences between low- and high-egg-laying hens, underscoring the essential regulatory networks that may support improved reproductive capacity.

### 3.4. Protein–Protein Interaction (PPI) Network

**Figure 6:**
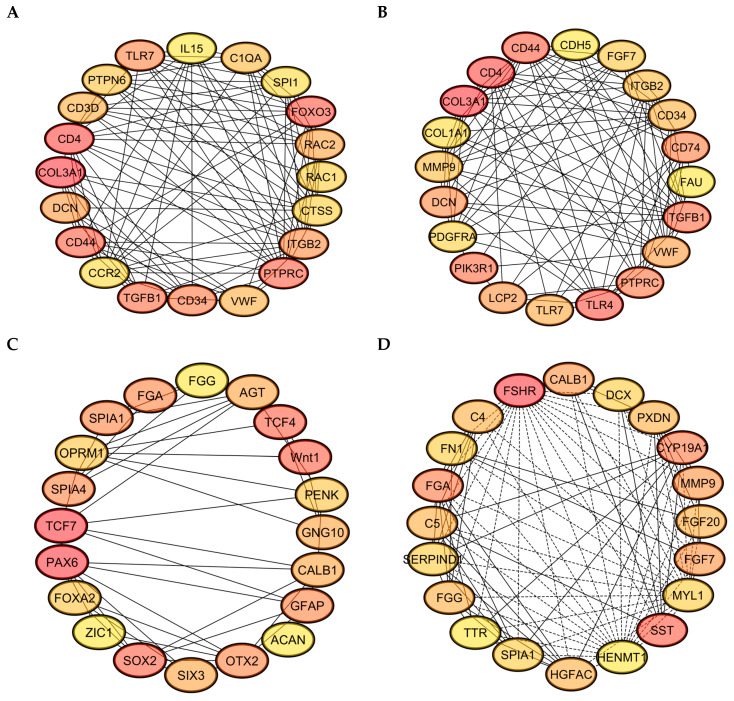
(**A**–**D**) The protein–protein interaction (PPI) networks of differentially expressed genes across four follicular groups were constructed utilising STRING and visualised through Cytoscape 3.10.3: (**A**) includes key genes like FOXO3, IL15, and CD4, which are implicated in immune regulation and stress response; (**B**) identifies TGFB1, CD44, and COL3A1, indicating their roles in cell adhesion and the organization of the extracellular matrix; (**C**) includes TCF4, TCF7, WNT1, and SOX2, indicating active Wnt signaling and transcriptional regulation; (**D**) includes hub genes such as CYP19A1, FGF7, and SST, which have essential roles in hormone biosynthesis and local growth factor activity, highlighting the dynamic regulatory mechanisms present in low and high egg-laying hens.

Analysis of the protein–protein interaction (PPI) network for differentially expressed genes linked to KEGG pathways across the four follicular stages—PRF, PMF, SWF, and SYF—revealed dynamic, stage-specific interaction patterns, underscoring essential regulatory mechanisms in folliculogenesis. During the initial phases (PRF and PMF), the networks showed significant enrichment for immune-related and cell-signaling genes, including CD4, CD44, TLR7, TGFB1, and VWF. This suggests a substantial role of immune modulation, cell adhesion, and extracellular matrix remodeling in the activation of early follicles. These genes are likely fundamental in the initiation of follicular growth and recruitment (Figure 6A–D).

During the intermediate and later stages, characterised by SW and SYF, the gene networks evolved to encompass those associated with hormone signalling, cellular differentiation, and oocyte maturation, including FGF7, CALB1, FSHR, and SST. The presence of transcription factors and developmental regulators, including PAX6, SOX2, and ZIC1, suggests that they have active transcriptional programming during the follicle transition and selection process. The connectivity and centrality of these hub genes within their networks indicate their potential role as key regulators of stage-specific biological processes. The progressive reorganisation of PPI networks across follicle stages suggests the temporal coordination of immune signalling, growth factor activity, and developmental control, potentially explaining the differences in reproductive capacity observed between high- and low-egg-laying hens.

### 3.5. RT-qPCR Validation

**Figure 7:**
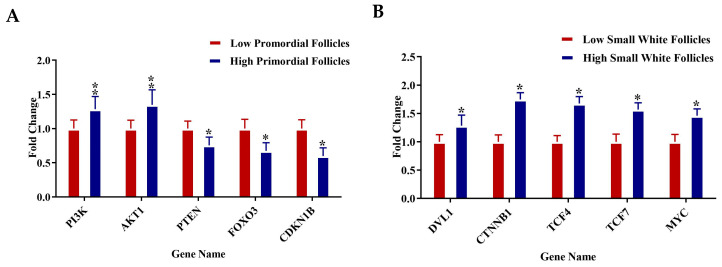

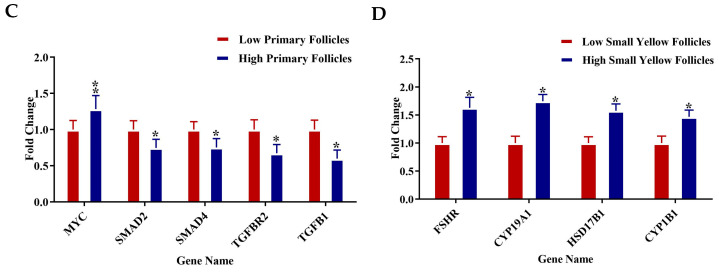
(**A**–**D**) Relative mRNA expression levels for specific genes associated with key regulatory pathways were examined in (**A**) PRF, (**B**) PMF, (**C**) SWF, and (**D**) SYF. Each panel contrasts gene expression between low (red bars) and high (blue bars) egg-laying hens. Genes linked to the PI3K-AKT, TGF-β, WNT, and steroidogenesis pathways exhibited notable differential expression throughout follicle stages. Expression values were set against internal controls, normalised to internal controls, and presented as fold change (mean ± SEM). Asterisks (*) denote statistically significant differences between groups (* *p* < 0.05; ** *p* < 0.01).

RT-qPCR analysis was conducted on selected genes across four follicle types—PR, pPM, SW, and SYF—to validate the transcriptome data and investigate key regulatory pathways related to follicular development in low- and high-egg-laying hens. In PRF, genes associated with the PI3K-AKT signaling pathway, such as PI3K, AKT1, PTEN, FOXO3, and CDKN1B, exhibited significantly elevated expression levels in low-egg-laying hens relative to high-egg-laying hens, indicating a potential inhibitory function in the activation of early follicles. In PMF, components of the TGF-β pathway, such as MYC, SMAD2, SMAD4, TGFBR2, and TGFB1, exhibited upregulation in low-egg-laying hens, suggesting a modification in the regulation of early follicular growth. Conversely, genes associated with the WNT signaling pathway, including DLL1, CTNNB1, TCF4, TCF7, and MYC, exhibited significant upregulation in the SWF of high-egg-laying hens, indicating increased WNT activity during this transitional phase. SYF from high-egg-laying hens exhibited increased expression of key steroidogenic genes, including FSHR, CYP19A1, HSD17B1, and CYP11B1, which aligns with the enhanced steroidogenic capacity necessary for follicle selection and maturation. The observed expression patterns reveal distinct pathway activation profiles at various stages of folliculogenesis, which may explain the reproductive performance disparities between low- and high-egg-laying hens (Figure 7A–D).

## 4. Discussion

This research demonstrates a correlation between high-egg-laying capacity in hens and specific molecular patterns throughout follicular development. Initial phases in high-performing hens indicate a transition from immune-centric signaling to improved metabolic and hormonal pathways, supported by significant genes in the PI3K-AKT, TGF-β, and WNT pathways. The findings corroborate earlier research, demonstrating that effective folliculogenesis necessitates precise stage-specific regulation of immune, metabolic, and steroidogenic processes. Molecular processes control egg production in ovarian follicles [26]. Optimising poultry reproduction requires understanding molecular differences between high- and low-egg-laying hen follicles [27]. From PRF to SYF, ovarian folliculogenesis exhibits distinct transcriptional patterns that influence egg production and function [28,29]. Gene regulation and egg-laying abilities remain poorly understood, despite considerable follicular development [29,30]. Integrating transcriptomic analysis across follicle phases to uncover regulatory pathways in both low- and high-egg-laying hens helps fill this gap. Molecular differences between follicle phases and egg-laying performance groups may explain reproductive results [31]. We performed the transcriptomes of PR, PM, SW, and SYF for DEGs associated with growth, differentiation, and maturity.

A Venn diagram revealed that low and high-laying hens possess distinct genes, indicating that these differences may influence egg-laying regulation. GO enrichment showed that low and high egg-laying chickens regulate cellular processes, metabolic regulation, and signaling pathways differentially across follicular phases. Heatmap analysis clustered follicle gene expression differences by growth and egg-laying performance. PCA showed that low and high egg-laying hens’ follicle transcriptional patterns differ along key elements, suggesting the transcriptome controls reproduction. In follicular development, immunological communication, metabolism, and hormone regulation were important [32]. Cytokine–cytokine receptor interactions and antigen processing are highly concentrated in PRF and PMF, indicating that immunological signaling activates these processes [33]. Steroidogenesis and cell differentiation are more common in SW and SY follicles [34]. High-laying hens have more pathways that range from immunological modulation to metabolic and hormonal regulation of follicle growth [35].

GO and KEGG enrichment analyses indicated that high-egg-laying hens showed significant enrichment in pathways associated with cell growth, hormone synthesis, and metabolic regulation, suggesting improved follicular functionality and reproductive efficiency. Biological processes, including granulosa cell proliferation, cell differentiation, lipid metabolic processes, and hormone biosynthetic processes, were significantly upregulated, indicating the heightened metabolic and endocrine activity necessary for follicle recruitment and ovulation. The enriched molecular function (MF) terms in the high-yield group, including steroid hormone receptor activity, oxidoreductase activity, and ATP binding, underscore the active steroidogenic and energy-regulating mechanisms present in the ovary. The cellular component (CC) terms, such as mitochondrial matrix, endoplasmic reticulum, and extracellular region, align with locations of hormone synthesis and secretion. KEGG enrichment analysis at the pathway level indicated significant overrepresentation of the PI3K-AKT, TGF-β, Wnt, and ovarian steroidogenesis pathways in high-laying hens, implying a coordinated regulatory network that promotes follicular development. The pathways are linked to cell survival, proliferation, differentiation, and hormonal responsiveness, ensuring the ongoing recruitment and maturation of follicles. In contrast, low-egg-laying hens exhibited an enrichment of immune-related pathways, including cytokine–cytokine receptor interaction, antigen processing and presentation, and Toll-like receptor signaling. This may indicate increased immune activation and cellular stress, which could hinder follicular growth. The differential enrichment profiles indicate that high reproductive efficiency correlates with a molecular transition from immune-dominant signaling in low-yield hens to metabolic, proliferative, and steroidogenic dominance in high-yield hens. The reprogramming of ovarian transcriptional landscapes offers a mechanistic explanation for the increased folliculogenesis and hormonal output seen in prolific layers.

**Figure 8:**
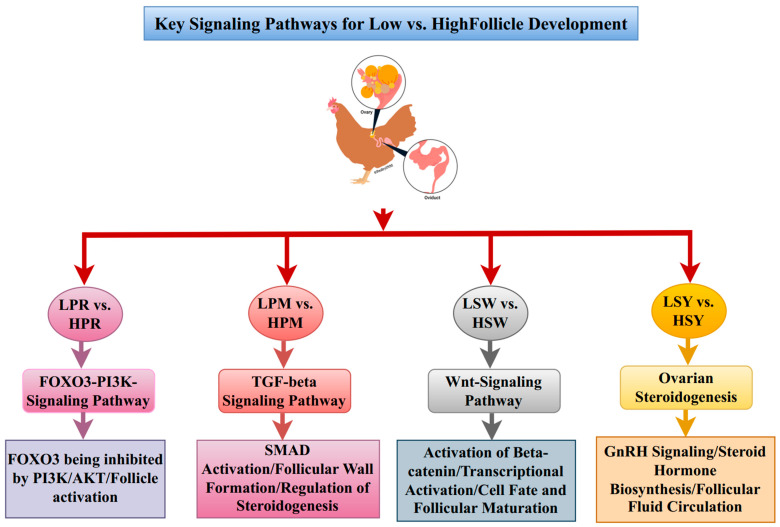
Schematic representation of key signaling pathways involved in the development of chicken follicles. The FOXO3-PI3K signaling pathway, TGF-beta signaling pathway, Wnt signaling pathway, and ovarian steroidogenesis pathway collectively regulate follicle development through four distinct stages: PR, PM, SW, and SYF. Pathway activation is represented in different colors with dynamic changes noted between low and high follicle stages.

A protein–protein interaction study on the complex molecular linkages that drive follicular growth (PPI) network revealed genes involved in immunological regulation, stress response, and hormone signaling [36,37,38]. These PPI networks demonstrated that folliculogenesis initially involves immunological regulation and cell adhesion, followed by hormone signaling and transcription factors [39,40]. The WNT and steroidogenesis pathways exhibited higher expression in high-laying chicken follicles, indicating their necessity for selection and maturation processes (Figure 8).

### 4.1. PI3K-AKT-FOXO3 Signaling: A Pivotal Axis in Primordial Follicular Activation

**Figure 9:**
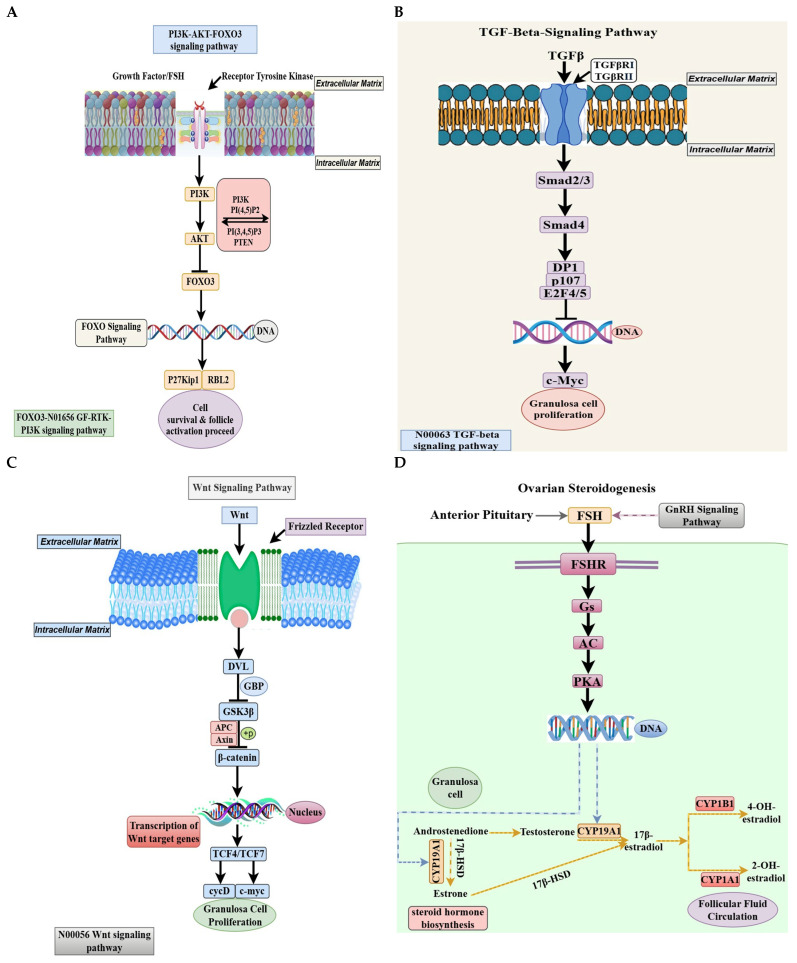
(**A**–**D**). (**A**) Schematic representation of signaling pathways in ovarian follicles of low and high-egg-laying hens. The PI3K-AKT-FOXO pathway activates PRF. Growth hormones and FSH activate receptor tyrosine kinases, leading to PI3K activation and subsequent phosphorylation of AKT. FOXO3 is unable to translocate to the nucleus in the presence of activated AKT. The pathway influences downstream targets such as P27^Kip1 and RBL2, thereby facilitating cell survival and follicle activation. In low-lying chickens, the expression of this pathway gene was elevated, indicating an inhibition of follicle activation. (**B**) The TGF-β pathway plays a vital role in PMF growth. Upon binding of TGF-β ligands to TGFBR1 and TGFBR2 receptors, a complex of Smad2/3 and Smad4 is formed, which subsequently translocates to the nucleus. This complex regulates cell cycle genes, including DP1, p107, E2F4/5, and c-Myc, to promote granulosa cell growth. Low- and high-laying hens exhibited differential gene expressions in this pathway, indicating its role in regulating early follicular growth. (**C**) In hens exhibiting low and high egg-laying capacity, SW and SY follicles demonstrate a high concentration of WNT signaling and ovarian steroidogenesis pathways. The WNT signaling cascade in SWF initiates with the interaction of WNT ligands with Frizzled receptors, leading to the activation of DVL and the inhibition of the GSK3β-APC-Axin complex. This mechanism stabilizes and translocates β-catenin into the nucleus. In the nucleus, β-catenin activates WNT target genes such as c-Myc and cyclin D in conjunction with TCF4/TCF7, thereby promoting granulosa cell growth. High-laying chickens exhibited upregulation of genes in this pathway, suggesting an enhancement in follicular transition and growth. (**D**) FSH binds to its receptor FSHR, initiating the Gs/AC/PKA signaling pathway, which transcriptionally activates essential steroidogenic enzymes in SYF. Enzymes such as CYP19A1, CYP17A1, HSD17B1, and CYP1B1 facilitate the conversion of cholesterol-derived androgens into estrogens, including oestrone, 17β-estradiol, and hydroxylated estrogens metabolites. The increased expression of these genes in prolific egg-laying hens suggests that steroidogenic activity plays a crucial role in follicle selection and ovulation.

Transcriptomic analysis of PRF showed a significant enrichment of the PI3K-AKT-FOXO3 pathway in both low- and high-egg-laying hens [41], with notable upregulation observed in high-laying birds [42]. Follicle-stimulating hormone (FSH) and growth factors stimulate phosphoinositide 3-kinase (PI3K) through receptor tyrosine kinases, facilitating the conversion of phosphatidylinositol 4,5-bisphosphate (PIP2) to phosphatidylinositol 3,4,5-trisphosphate (PIP3), whereas phosphatase and tensin homolog (PTEN) restricts this conversion [43,44]. Increased AKT phosphorylation in high-laying hens suggests a potential decrease in PTEN activity or an enhancement of PI3K signaling [45]. Activated AKT phosphorylates FOXO3, leading to its nuclear export and transcriptional inactivation, thereby promoting genes associated with cell survival, proliferation, and follicular growth [46,47]. Nuclear FOXO3 inhibits follicular activation by suppressing cyclin D2, RBL2, and P27Kip1, thereby maintaining oocyte dormancy [46,48]. High-laying hens exhibit decreased nuclear FOXO3 targets, suggesting enhanced follicular activation and atresia [49]. The findings indicate a molecular environment conducive to early follicle development and ovulation, providing essential insights into avian ovarian regulation and potential targets for enhancing reproductive efficiency in poultry (Figure 9A).

The integration of the PI3K-AKT-FOXO3 pathway with other regulatory networks, including TGF-β, WNT, and mTOR, influences granulosa cell proliferation, oocyte development, and follicle dynamics [50,51]. The transcriptome profiles of high-laying hens show synergistic activation of several signalling pathways, revealing a coordinated molecular program that facilitates folliculogenesis [52]. The PI3K-AKT pathway promotes granulosa cell survival by interacting with anti-apoptotic signals [53]. Modulating PI3K-AKT-FOXO3 influences poultry breeding and productivity [54]. The differential expression patterns revealed in this study may help breeders identify hens with more advanced intrinsic follicular development.

Transcriptomics reveals dynamic gene expression; however, the critical roles of folliculogenesis necessitate CRISPR/Cas9 editing, in vitro granulosa cell models [55], or in vivo overexpression and silencing techniques [56]. The expression of PTEN, AKT phosphorylation, and nuclear localisation of FOXO3 elucidate the PI3K-AKT-FOXO3 pathway. Recent studies have highlighted the roles of epigenetic regulators and non-coding RNAs, suggesting that polymorphisms in PTEN, FOXO3 lncRNA, or miRNA may provide insights into follicular control [57]. The PI3K-AKT signaling pathway enhances the survival and proliferation of follicular cells, processes that are disrupted in low-laying hens and likely contribute to their diminished reproductive efficiency. Poultry breeding objectives must encompass both genetic selection and physiological regulation to achieve reproductive success through intracellular signaling.

### 4.2. TGF-β Signaling Orchestrates Granulosa Cell Proliferation in Folliculogenesis

TGF-β signaling is crucial for ovarian folliculogenesis, regulating granulosa cell proliferation, oocyte maturation, and follicular development [58,59]. We examined the expression of TGF-β pathway components in PMF of low- and high-egg-laying hens. This pathway plays a role in reproductive differences, as it is transcriptionally active and differentially regulated across performance groups. TGF-β activates its cell surface serine/threonine kinase receptors, TGFβRI and TGFβRII, upon ligand binding, initiating a series of intracellular processes [60]. The increased transcription of both receptor subunits in high-egg-laying hens suggests enhanced TGF-β signaling sensitivity during their follicular growth. This is important for downstream SMAD-dependent signaling, particularly involving SMAD2/3 and the shared mediator SMAD4 [61]. Upon activation, SMAD2/3 undergoes phosphorylation and assembles heteromeric complexes with SMAD4, subsequently translocating to the nucleus to modulate gene expression [62]. High-laying hens exhibited high levels of SMAD2, SMAD3, and SMAD4 transcripts, suggesting enhanced TGF-β signaling in PM follicle granulosa cells [63,64].

This indicates enhanced transcriptional activation of genes involved in cell cycle and proliferation. Data show that SMAD complexes interact with transcriptional co-activators such as DP1, p107, and the E2F family (E2F4/5), which are involved in regulating the G1/S transition in proliferating cells [65]. The high expression of downstream nuclear effectors in high-egg-laying hens indicates a correlation between TGF-β signalling and the regulation of follicle development and granulosa cell proliferation (Figure 9B). The overexpression of the proto-oncogene c-Myc, which promotes cellular proliferation and metabolic activity, supports this finding within the same group [66].

Oocytes depend on granulosa cells for paracrine and metabolic support during follicular development [67]. Ovulation is increased by granulosa cell proliferation, which increases follicular size and estradiol production [68]. Data show that activating the TGF-β pathway leads to enhanced mitotic activity in granulosa cells of high-laying hens, as demonstrated by increased transcription of proliferation-related genes and signaling pathways [69]. However, low-laying hens showed lower expression of these components, indicating weaker TGF-β signaling [70]. TGF-β affects FOXO transcriptional activity and cell viability by modulating the PI3K-AKT signaling pathway through non-canonical pathways [71]. In high-laying hens, these networks are upregulated simultaneously, indicating a coordinated signaling environment that prepares follicles for activation, survival, and growth [72]. The TGF-β pathway emphasizes E2F transcription factors, which are downstream targets of AKT signaling, as they are phosphorylated by RB-related proteins [73].

TGF-β signaling is crucial for vertebrate folliculogenesis, promoting the transition from primary to secondary follicles and facilitating communication between oocytes and granulosa cells in chickens [74]. Transcriptome analysis identifies regulatory elements; however, confirmation through immunohistochemistry, in situ hybridization, and single-cell transcriptomics is necessary [75]. Functional studies of TGF-β receptors and SMADs will demonstrate their causal involvement. Investigating miRNAs, lncRNAs, and epigenetic factors may reveal new regulatory mechanisms. TGF-β promotes granulosa cell proliferation and follicular development through the PI3K-AKT signaling pathway, offering insights into avian reproduction and potential advancements in chicken breeding.

### 4.3. The Wnt Signaling Pathway: A Crucial Mediator of Granulosa Cell Proliferation

Transcriptomic analysis of SWF in chickens with varying egg-laying capacity reveals that the Wnt signaling pathway regulates follicular growth and egg-laying performance. Cell proliferation, fate determination, and differentiation, notably in the ovary, are closely related to the Wnt pathway [76,77]. High-egg-laying chickens exhibit increased expression of the β-catenin-dependent Wnt signaling pathway, indicating its direct role in boosting granulosa cell proliferation, follicle transition, and higher reproductive output [76,78]. The canonical Wnt pathway begins when Wnt ligands engage with Frizzled (Fz) receptors and co-receptors on granulosa cells [79,80]. High-laying chickens had higher transcription of Wnt ligands and Frizzled receptors, suggesting they can initiate Wnt signal transduction (Figure 9C). The ligand–receptor contact activates Dishevelled (DVL) proteins and scaffolding proteins, such as GBP, which inhibit the β-catenin degradation complex comprising Axin, APC, and GSK3β [81,82].

After activating canonical Wnt signaling, β-catenin stabilizes and accumulates in the cytoplasm before influencing gene transcription in the nucleus [83]. Increased β-catenin (Figure 6C) and decreased negative regulators (GSK3β, APC, and Axin) in high-laying chickens create a favourable molecular environment for protein stability. GSK3β downregulation or post-transcriptional suppression, which phosphorylates β-catenin for proteasomal degradation, results in its accumulation [84]. After entering the nucleus, β-catenin activates Wnt gene transcription by interacting with TCF4/TCF7 transcription factors [85]. Cell cycle progression and proliferation require cycD and c-Myc [86,87]. High-egg-laying hens regularly upregulate cycD and c-Myc, which increase granulosa cell mitosis and follicular expansion for early development and frequent ovulation [88]. Increased Wnt signaling in high-laying chickens prepares follicular cells for FSH-dependent selection and ovulation [89]. Hens laying lower eggs have lower β-catenin levels, reduced granulosa cell proliferation, fewer preovulatory follicles, and lower egg production [90].

The Wnt signalling pathway is integral to ovarian physiology, interacting significantly with the PI3K-AKT and TGF-β pathways [91]. In high-laying hens, SMAD proteins from the TGF-β pathway may interact with β-catenin and TCF4 to augment granulosa cell gene expression in response to both internal and external signals [92]. A feedback loop involving Wnt/β-catenin and AKT signalling facilitates the survival and proliferation of granulosa cells [93]. Wnt activation significantly increases the expression of c-Myc and cyclin D1 (cyclin D; cycD), which are essential for ribosome biogenesis, metabolic processes, DNA replication, and progression of the cell cycle from G1 to S phase [94]. This transcriptional signature indicates increased granulosa cell activity, facilitating follicular expansion, estrogen synthesis, and regular ovulation [95]. In contrast, low-laying hens demonstrate decreased expression of c-Myc and cycD, resulting in granulosa cell quiescence and restricted follicular growth [96]. The findings indicate the coordinated activation of signaling networks that facilitate high reproductive output in prolific egg-laying chickens.

Unlike in mammals, Wnt signaling regulates PRF activation, selection, and luteinisation in birds [90]. Follicular reserves and egg production in hens depend on precise spatial and temporal regulation of Wnt activity [97]. Elevated β-catenin, TCF4, or cyclin D1 levels in follicles indicate reproductive potential and serve as selection markers [98]. Nutrition, hormones, or gene editing can modulate Wnt signalling to recruit follicles; however, overactivation can lead to aberrant proliferation, necessitating precise management [99]. Although transcriptome evidence suggests Wnt involvement, further validation through protein-level investigations and functional experiments using RNAi or CRISPR-Cas9 is necessary [100]. Single-cell and spatial transcriptomics can identify responsive granulosa or theca subpopulations and canonical pathways [101]. Transcriptomic analysis of SWF reveals Wnt/β-catenin signaling as a significant regulator of granulosa cell proliferation and early activation, identifying potential targets for genetic selection and reproductive improvement.

### 4.4. Ovarian Steroidogenesis: A Hormonal Engine of Follicular Maturation

The production of steroid hormones is essential for regulating folliculogenesis, ovulation, and egg-laying in chickens [102]. Follicle-stimulating hormone (FSH) promotes the expression of steroidogenic enzymes in the granulosa cells of developing follicles [103]. Transcriptomic analysis of SYF, which are essential for recruitment and selection, demonstrates distinct gene expression patterns in low- versus high-egg-laying hens [104,105]. In high-laying birds, the expression of the FSH receptor (FSHR) is elevated, suggesting increased responsiveness [106]. The upregulation of downstream components, including Gs proteins, adenylyl cyclase (AC), and protein kinase A (PKA) subunits, enhances the efficacy of intracellular FSH signaling [107]. The molecular characteristics enhance the transcriptional activation of steroidogenic genes in granulosa cells, particularly those related to estrogen synthesis [108,109]. The observed differences offer significant insights into hormonal regulation that enhances reproductive performance in high-egg-laying hens (Figure 9D).

The FSH/PKA pathway modulates CYP19A1, which encodes aromatase, an enzyme responsible for converting androgens to estrogens, such as testosterone to estradiol [110]. Transcriptomic data indicate that high-laying hens demonstrate increased CYP19A1 expression in SYF, associated with heightened FSH signaling and PKA activation [111]. This enhances estrogen production, facilitating follicular maturation, granulosa cell proliferation, vitellogenin synthesis, and oviductal preparation [112]. Furthermore, elevated levels of 17β-hydroxysteroid dehydrogenase (17β-HSD) in high-laying hens indicate an enhanced conversion of steroid precursors, such as androstenedione and estrone, into active hormones, including testosterone and estradiol [113]. The balanced enzymatic activity fosters a favourable hormonal environment that enhances follicular growth and recruitment into the pre-ovulatory hierarchy, thereby improving reproductive efficiency in prolific egg-laying chickens [114].

Estradiol metabolism and synthesis are crucial in regulating estrogenic activity and maintaining follicular fluid balance [115,116]. High-laying hens demonstrate elevated expression of CYP1A1 and CYP1B1, enzymes that hydroxylate estradiol into less active metabolites, such as 2-hydroxyestradiol [117]. This indicates active estradiol turnover, potentially mitigating hormonal overstimulation and atresia. The SYF exhibit coordinated upregulation of essential steroidogenic genes, including FSHR, CYP19A1, and 17β-HSD, as well as metabolic enzymes [118,119], which support follicular competence and the transition to hormone-dependent growth. This molecular profile enables efficient recruitment and selection of follicles into the pre-ovulatory hierarchy. In contrast, low-laying hens exhibit diminished expression of these genes, suggesting reduced responsiveness to FSH and decreased estradiol synthesis, resulting in a lower number of competent follicles reaching ovulation [120].

GnRH signalling modulates FSH secretion from the anterior pituitary, coordinating systemic endocrine signals with ovarian requirements [121,122]. In high-laying hens, enhanced activity of the HPG axis results in elevated FSH levels and influences follicular gene expression, thereby facilitating coordinated follicular growth [123]. Pituitary transcriptomics and plasma hormone profiling elucidate this regulatory axis, which involves the specific regulation of steroidogenesis due to the hen’s rapid ovulatory cycle [124]. High egg-producing hens improve the expression of steroidogenic genes in SYF, thereby optimizing their development. FSHR, CYP19A1, and 17β-HSD are identified as molecular markers for selecting high-performing layers [125]. Targeting transcriptional and hormonal pathways may enhance reproduction through nutritional or pharmacological interventions; however, overstimulation can lead to follicular pathologies. Validation through protein assays, enzyme activity, and hormonal profiling is essential, in combination with functional studies in granulosa cells or in vivo. Transcriptome data indicate the upregulation of genes such as CYP1A1 and CYP1B1, which facilitate estradiol metabolism, granulosa cell proliferation, and ovulation. Molecular traits improve the endocrine environment and represent potential targets for genetic selection in poultry breeding.

The PI3K-AKT-FOXO3, TGF-β, Wnt, and ovarian steroidogenesis pathways form a cohesive regulatory network that regulates follicular activation, granulosa cell proliferation, and hormonal responsiveness in hens exhibiting varying egg-laying capacities. The PI3K-AKT-FOXO3 axis triggers the activation of primordial follicles by influencing cell survival and the dormancy of oocytes. In contrast, TGF-β signaling regulates the proliferation of granulosa cells via SMAD-dependent transcriptional mechanisms. Simultaneously, the Wnt/β-catenin pathway enhances signals for proliferation and differentiation, working in conjunction with both PI3K-AKT and TGF-β signaling to support follicular transition and growth. Following these molecular pathways, ovarian steroidogenesis converts intracellular signaling into hormonal outputs that govern follicle selection and ovulation. The interactions among these pathways create a molecular continuum—from follicular activation to steroid-driven maturation—that explains the enhanced reproductive performance of high-egg-laying hens. Gaining insights into these integrated networks is crucial for enhancing genetic selection, reproductive management, and overall productivity in poultry.

## 5. Conclusions

This research presents transcriptomic evidence indicating that variations in egg-laying performance are associated with different gene regulatory networks throughout follicular stages. Data show that high productivity correlates with the coordinated upregulation of essential pathways, including PI3K-AKT, TGF-β, and Wnt/β-catenin, which may facilitate follicular growth and cell proliferation. The increased expression of steroidogenic genes such as CYP19A1 and FSHR in high-laying hens indicates a more efficient production of hormones. These findings identify potential molecular markers of reproductive efficiency that may inform future genetic selection strategies in poultry breeding.

## Figures and Tables

**Table 1:** Egg-laying performance of low- and high-egg-laying chickens of the Xinghua breed.

Group	Number of Hens (*n*)	Mean Eggs Laid (Mean ± SD)
Low Egg Laying	*n* = 12	58.83 ± 8.05
High Egg Laying	*n* = 12	121.17 ± 5.98

## Data Availability

The datasets generated and analyzed during the current study are available in the form of Supplemental Tables S1–S19.

## Supplementary Materials

The following supporting information can be downloaded at: https://www.mdpi.com/article/10.3390/ani15223300/s1, The datasets generated and analyzed during the current study are available as Tables S1–S19.

## Abbreviations

The following abbreviations are used in this manuscript:

PRF	Primordial follicles
PMF	Primary follicles
SWF	Small white follicles
SYF	Small yellow follicles
