# Supplementary material for: Integrative Transcriptome Analysis Across Follicles Highlights Key Regulatory Pathways in Low and High-Egg-Laying Hens
Source: Animals (Basel). 2025 Nov 15;15(22):3300. doi: 10.3390/ani15223300 (PMC12649194; doi:10.3390/ani15223300)
Supplement: Supplementary file 1 [file animals-15-03300-s001.zip › Table_S18_RT_PCR_Genes.pdf]

No.	Gene	ID	Primer	bp
1	AKT1	>NM_001396388.1	CCACACGCTTTCTGAGCAGC<br>GTCTTGCGGTCGTTCCTTGT	531
2	MTOR	>XM_417614.8	CGAATCTGTAACGCTCCGCC<br>TCCTGGCTCATTTCACGGAG	749
3	RPS6KB1	>XM_046930143.1	CCTCTCCCAGGCTTTAAGGAA<br>TGGAGATGGGTGAGCAAACG	469
4	PTEN	>XM_040674795.2	GCAGCCATGATGGGAGCGTA<br>ATTGCAAGTTCCGCCACTGA	743
5	CTNNB1	>XM_046910392.1	TCCAGGAATGAGGGTGTTGC<br>ATGATACAGCATCTGGGCGG	211
6	WISP1	>XM_046910739.1	CTGCAGCATATGAGGGGGAG<br>CCATCTGGGCACTCAAACCT	376
7	MAPK8	>XM_040703166.1	TGGTGCCAAACCAGATAGGG<br>GGATGACCTCTGGTGCTCTG	715
8	PCYT1A	>XM_046898487.1	GAGTGAACATGGCTGCACGA<br>TCCAAAGGGGTCCCTCTCAT	388
9	ETNK1	>XM_416426.8	TGTTCTGCACCAGGTGGTTT<br>GAACCCGAGACAAACGCTCT	212
10	RBL2	>NM_001397481.1	AGCACAGGTAGGCCCTTCTC<br>TGAGTGCCTCGGTACAATGC	446
11	CDKN1B	>NM_204256.3	CTGGAAGGCAGGTACGAGTG<br>TCCTCCGCCTTAGGGAGTTT	408
12	FOXO3	>XM_001234495.7	CTGGCACACCCCAAAATGTG<br>CGCTGTGGACATCACCCAT	149
